# Representation of images of black skin in pediatric textbooks^☆^

**DOI:** 10.1016/j.abd.2024.07.006

**Published:** 2024-11-27

**Authors:** Maria Eduarda Duarte de Oliveira, Ronald Godinho de Oliveira Silva, Melissa Moreira Mansur Clemente, Sabrine Teixeira Ferraz Grunewald

**Affiliations:** Faculty of Medicine, Universidade Federal de Juiz de Fora, Juiz de Fora, MG, Brazil

Dear Editor,

Dermatological conditions are highly prevalent in children, being a complaint in approximately 30% of pediatric consultations.^1^ They can be manifestations of systemic diseases, such as fungal, viral, bacterial, parasitic infections, drug reactions, or genetic diseases, and an adequate physical examination is important to identify the type of lesion.

Skin manifestations present differently in black skin when compared to light colored skin, which when unknown to the physician leads to underdiagnosis, compromising patient prognosis due to a delayed or incorrect treatment.^2^ There are studies that have shown, for example, that black women are three times more likely to be underdiagnosed with systemic lupus erythematosus.^3^ Other dermatoses that are also commonly misdiagnosed include vitiligo, often considered a suspected case of leprosy, and pityriasis versicolor, confused with seborrheic dermatitis, erythrasma, secondary syphilis, or pityriasis rosea.^3^

In Brazil, the 2022 Census revealed that the majority of respondents declared themselves to be brown, representing 45.3% of the population, while the proportion of white individuals decreased and that of black, brown, and indigenous people increased compared to 2010.^4^ However, there is a lack of studies on the representation of black skin in Brazilian reference books, which motivated this research. Thus, we evaluated the images found in pediatric textbooks, verifying the frequency of photographs portraying dermatological conditions in black skin.

For the study, three general pediatric textbooks and one pediatric dermatology textbook were selected. Three of them were available in the virtual library of Universidade Federal de Juiz de Fora, Minas Gerais state, Brazil, where the research was carried out, and a digital edition of the fourth book was purchased. The list of selected books and chapters is available in the Supplementary Material. These books were selected from among the books available in the virtual library because they showed color images, unlike the other books consulted.

The study included images of skin lesions available in the chapters related to dermatological or infectious diseases (e.g., exanthematous diseases). The images were extracted from the books and cataloged, after excluding those in black and white; those that exclusively showed mucous membranes, conjunctiva, palms of the hands or soles of the feet, which prevented the identification of the skin color of the patient.

Skin color was assessed by two independent researchers, according to the skin phototypes in the Fitzpatrick scale. Skin types I to III were considered light skin, and types IV to VI were considered dark skin, as previously defined in the literature.^5^ Discrepancies between the researchers were resolved by a third author. In the statistics, a descriptive analysis was performed, and interobserver agreement was assessed using Cohen's Kappa test.^6^

A total of 797 images met the inclusion criteria and were initially selected. Of these, 91 were subsequently excluded, as they did not allow an appropriate assessment of skin color: nine were black and white images; 79 exclusively displayed mucous membranes, conjunctiva, palms, or soles; two were repeated; and one represented a fetus. Therefore, 706 images were included, but 11 of them could not be classified due to image quality issues or because the photo displayed only the lesion, with no healthy skin to be evaluated. Thus, 695 images were classified according to skin color. When classifying the images as light skin or dark skin, the researchers achieved moderate interobserver agreement, with a Kappa of 0.704 and p < 0.001.

The absolute and relative frequencies of images depicting black and white skin are detailed in Table 1. The proportion of images of black skin varied between 16.3% and 37.7%, with book four showing the highest percentage. Regarding the diseases covered, pityriasis had the highest percentage of black skin images (53.5%), while rheumatological and immune diseases had the lowest percentage (14.1%).

**Table 1:** Representation of skin colors in the analyzed images, for each book and group of diseases.

	n (%)	
	Black skin	White skin
**Book 1 (n = 172)**	28 (16.3)	144 (83.7)
**Book 2 (n = 87)**	15 (17.3)	72 (82.7)
**Book 3 (n = 28)**	7 (25.0)	21 (75.0)
**Book 4 (n = 408)**	154 (37.7)	254 (62.3)

**Rheumatological/immunological (n = 85)**	12 (14.1)	73 (85.9)
**Acne (n = 25)**	4 (16.0)	21 (84.0)
**Diaper dermatitis (n = 5)**	1 (20.0)	4 (80.0)
**Other dermatites (n = 21)**	5 (23.8)	16 (76.2)
**Nail conditions (n = 12)**	3 (25.0)	9 (75.0)
**Exanthematous diseases (n = 46)**	12 (26.0)	34 (74.0)
**Elementary lesions (n = 31)**	9 (29.0)	22 (71.0)
**Congenital lesions (n = 128)**	38 (29.7)	90 (70.3)
**Skin infections (n = 132)**	40 (30.3)	92 (69.7)
**Dyschromias (n = 8)**	3 (37.5)	5 (62.5)
**Atopic dermatitis (n = 35)**	13 (37.1)	22 (62.9)
**Urticaria (n = 10)**	4 (40.0)	6 (60.0)
**Pityriases**^a^**(n = 15)**	8 (53.3)	7 (46.7)
**Others (n = 142)**	52 (36.6)	90 (63.4)

**Total (n = 695)**	204 (29.3)	491 (70.7)

a Includes, pityriasis, alba, rubra and lichenoid forms.

Fig. 1 shows a graph of the percentage of images classified as white or black skin for each book and in total. Fig. 2 shows the percentage of images classified as white or black skin according to the disease or dermatological lesion.

**Figure 1:**
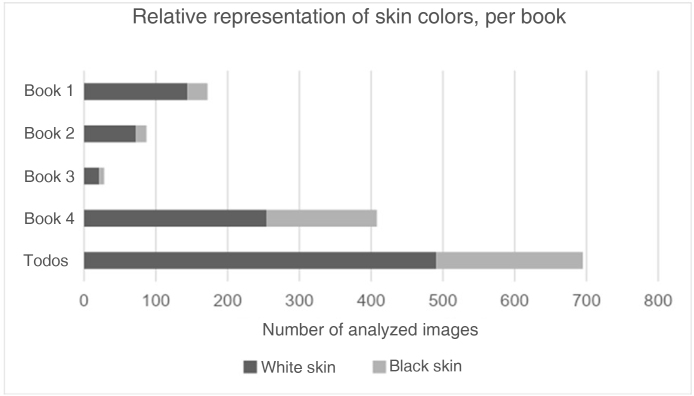
Number of images representing black and light skin in each book.

**Figure 2:**
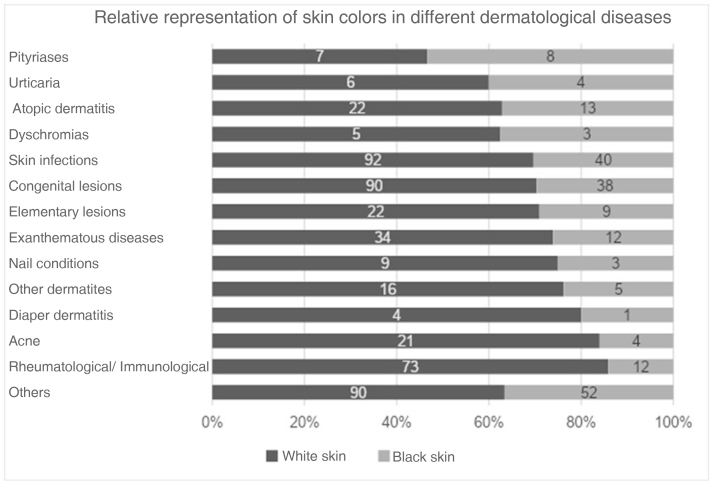
Number of images representing black and light skin in each group of dermatological diseases.

The ability to identify skin manifestations of diseases in different skin phototypes is essential in the evaluation and determination of the conduct by health professionals. The results of this research show that most of the images that illustrate the assessed pediatric and pediatric dermatology books depict skin lesions in white patients. This becomes a problem when one understands that, depending on skin color, the same disease can manifest itself in different ways.^5^

Research aimed at analyzing images in medical textbooks and materials had been conducted with the intention of ascertaining the representation of black skin. A survey of ophthalmology textbooks showed that 92.1% of the images were of light skin and 7.9% of dark skin,^7^ while similar research conducted by the American College of Rheumatology states that in rheumatology materials, only 13.4% of the images showed black skin.^8^

In addition, studies have highlighted a significant underrepresentation of black skin in medical education curricula in the United States, so that the lack of adequate instruction begins in the undergraduate course and persists throughout residency. This educational gap is evident in the scarcity of images that depict common and rare dermatological conditions in darker skin in teaching materials and educational resources, as well as the lack of formal training in many residency programs.^9^

A study showed that almost 50% of dermatologists did not have sufficient experience with the management of lesions on black skin during their training.^10^ This becomes a matter of concern, because ethnic minorities have worse outcomes from melanoma and non-melanoma skin cancer.^10^ Also, a study showed the lack of adequate representation of black skin in images related to skin manifestations of COVID-19, with direct consequences for public health, especially considering the disproportionate impact of the pandemic in the black community.^5^

As limitations of this study, we highlight the selection of books on pediatrics, which may limit the representation of more common skin lesions in adults. There is an interobserver subjectivity in the assessment of skin phototypes which may affect the accuracy of classifications. Furthermore, the fact that photographs were being analyzed represents a challenge, since the lighting and sharpness of the image may distort the perception of skin color. However, the systematic approach in the selection of books and images increases transparency and reproducibility. The use of the validated Fitzpatrick scale allows greater standardization in the classification, reducing subjectivity. The use of Cohen's Kappa test to assess interobserver agreement provides a robust measure of the consistency of the classification.

The underrepresentation of black patients with skin lesions in the scientific community not only reflects but also perpetuates disparities in the health care of patients of different races and ethnicities. The lack of a variety of images of dermatological conditions in black skin can result in lack of familiarity by healthcare professionals to identify and diagnose these conditions in patients with darker skin. This lack of knowledge can lead to delays, underdiagnosis, or misdiagnosis, impacting the quality and effectiveness of treatment. These negative consequences highlight the critical importance of increasing the representation and visibility of dermatological conditions in black patients in the scientific literature and clinical practice. Curricular changes to increase the recognition of diverse phototypes and the creation of imaging atlases and dermatology manuals focused on black skin are required.

## Financial support

None declared.

## Authors' contributions

Maria Eduarda Duarte de Oliveira: Collection of data; drafting and editing of the manuscript; critical review of the literature.

Ronald Godinho de Oliveira Silva: Collection of data; drafting and editing of the manuscript; critical review of the literature.

Melissa Moreira Mansur Clemente: Collection of data; drafting and editing of the manuscript; critical review of the literature.

Sabrine Teixeira Ferraz Grunewald: Design and planning of the study; statistical analysis; critical review of the literature; approval of the final version of the manuscript.

## Conflicts of interest

None declared.
