# Supplementary material for: Representation of images of black skin in pediatric textbooks
Source: An Bras Dermatol. 2024 Nov 27;100(2):346–9. doi: 10.1016/j.abd.2024.07.006 (PMC11962813; doi:10.1016/j.abd.2024.07.006)
Supplement: Supplementary file 1 [file mmc1.docx]

ABD-D-24-00377_Supplementary material

**Supplementary Material**

**Table 1** List of books and chapters from which images were extracted for research.

| **Number** | **Book** | **Chapters** |
| --- | --- | --- |
| 1 | Richard E. Behrman, Robert M. Kliegman, Hal B. Jenson. Nelson Textbook of Pediatrics. Elsevier, 21^st^ edition, 2019. | 170, 173, 273 to 284, 664 to 691 |
| 2 | Luciana Rodrigues Silva, Dirceu Solé. Diagnóstico em Pediatria (Volumes 3 e 4), Editora Manole, 2^nd^ edition, 2022. | 49 and 75 |
| 3 | Ligia Maria Suppo de Souza Rugolo et al. Pediatria: do Recém-nascido ao Adolescente. Editora Atheneu, 1^st^ edition, 2020. | 34, 37, 49, 50, 51, 52, 53 |
| 4 | Maria Cecilia da Matta Rivitti Machado. Dermatologia Pediátrica. Editora Manole, 3^rd^ edition, 2022. | 3 to 60 |
